# Three‐dimensional simulation analysis of microdissection testicular sperm extraction for patients with non‐obstructive azoospermia

**DOI:** 10.1111/andr.12812

**Published:** 2020-05-18

**Authors:** Kentaro Ichioka, Yoshiyuki Matsui, Naoki Terada, Hiromitsu Negoro, Takayuki Goto, Osamu Ogawa

**Affiliations:** ^1^ Ichioka Urological Clinic Kyoto Japan; ^2^ Department of Urology Kyoto University Graduate School of Medicine Kyoto Japan

**Keywords:** male infertility, microdissection, non‐obstructive azoospermia, simulation, TESE, testis

## Abstract

**Background:**

Microdissection testicular sperm extraction (microTESE) is considered the gold standard method of sperm retrieval from patients with non‐obstructive azoospermia (NOA). For careful and thorough examination of seminiferous tubules during microTESE, maximizing surface area of the testicles which we are able to search is essential.

**Objectives:**

To develop a systematic procedure for microTESE to maximize surface area and to achieve high sperm retrieval rate (SRR) in microTESE.

**Materials and methods:**

We simulated microTESE using three‐dimensional (3D) simulation model and analyzed mathematically the sum of the surface area in various methods. The best method obtained from this simulation model was applied to 102 patients with NOA from 2014 to 2018. These new clinical results were compared with those of 56 patients who underwent a previous method of microTESE from 2011 to 2014.

**Results:**

The mathematical 3D simulation model of microTESE indicated that a longitudinal incision on the tunica albuginea and following transverse slicing incisions of testicular parenchyma maximized the surface area coverage. Forty‐six (45%) out of 102 patients who underwent microTESE with the new method had successful retrieval of testicular spermatozoa compared with 16 (29%) of 56 patients with the previous method of microTESE (*P* = .04).

**Discussion:**

Transverse resections of parenchyma in our method run parallel to the courses of intratesticular arteries and do not interfere with the blood supply. The small amount of extracted seminiferous tubules was equivalent to that of the previous method, and no patients exhibited post‐operative symptoms of androgen deficiency in our study. As for post‐operative pain, our new method was comparable with the previous method. Although our study needs a longer follow‐up, there will be limited effects on testicular functions.

**Conclusion:**

Longitudinal incision on the tunica albuginea and following transverse slicing incisions in the testicular parenchyma maximized the surface area and improved the SRR of microTESE.

## 1 INTRODUCTION

MicroTESE is considered the gold standard method of sperm retrieval from patients with NOA. It is well known that the dilated, white seminiferous tubules are more likely to have intact spermatogenesis and that the use of surgical microscope to search suitable tubules benefits the successful retrieval of spermatozoa in NOA patients.^1^ However, despite careful and thorough examination of seminiferous tubules during microTESE, there is still a possibility that we may overlook suitable tubules and come to a wrong conclusion. Several previous papers concerning repeat microTESE have reported the possibility of sperm retrieval in cases of previous failed surgery.^2^, ^3^, ^4^, ^5^ For the patients to accept the final outcomes, regardless of whether it is hopeful or not, we need to maximize the area of the testicles which we are able to search.

Generally, microscopic surgery is technically demanding and needs advanced training. Ishikawa et al reported that the surgical outcomes and SRR of microTESE performed by one surgeon will improve with experience and showed substantial learning curves for microTESE.^6^ However, there is no standardized idea to search for suitable tubules during microTESE. Furthermore, there is controversy about the types of initial incision for microTESE, transverse or longitudinal, with supporters on both sides.^7^ Thus, we need to develop a standardized, systematic procedure for microTESE to minimize the possibility of overlooking suitable tubules and sperm retrieval.

Here, we used 3D simulation of microTESE to develop the best method to maximize the surface area of cut planes and systematically check all the seminiferous tubules of testicular parenchyma. Also, we applied this new method to recent NOA patients, to demonstrate its safety and improvement of surgical outcomes for the rate of successful sperm retrieval.

## 2 MATERIALS AND METHODS

### 2.1 3D simulation of microTESE

**Figure 1:**
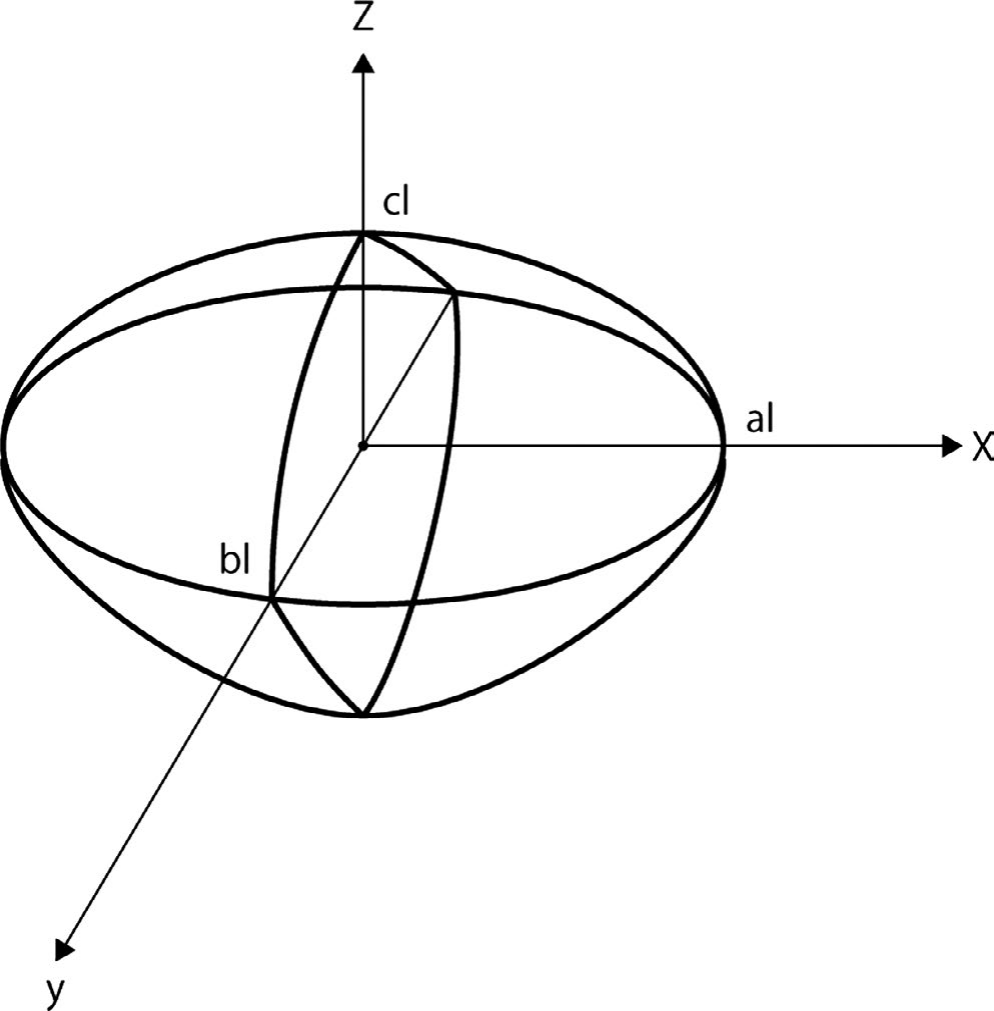
Mathematical model of the testis, which has an ellipsoid shape with the lengths of each axis denoted as *Al*, *Bl,* and *Cl*, with *Al* being the longest and *Cl* the shortest (Al > Bl>Cl). This testis model was placed into the X‐Y‐Z axis with the longitudinal incision parallel to the X‐axis and the coronal and sagittal planes corresponding to the X‐Y and X‐Z planes, respectively

We assumed that the testis had an ellipsoid shape with the lengths of each axis denoted as *A*, *B,* and *C*, with *A* being the longest and *C* the shortest (A > B>C). We placed this testis model into the X‐Y‐Z axis, where the longitudinal incision was parallel to the X‐axis and the coronal and sagittal planes were equivalent to the X‐Y and X‐Z planes, respectively. After a long incision on the tunica albuginea, testicular parenchyma was divided into several thin‐sliced pieces in order to fully search the testicular contents. The width of a sliced piece of testicular parenchyma was assumed to be *l*, and we assumed *A = al*, *B = bl,* and *C = cl* (Figure 1). This testis model could be described as follows:

$$\frac{\begin{array}{c} x^{2} \end{array}}{\begin{array}{c} {\left(\mathrm{al}\right)}^{2} \end{array}}+\frac{\begin{array}{c} y^{2} \end{array}}{\begin{array}{c} {\left(\mathrm{bl}\right)}^{2} \end{array}}+\frac{\begin{array}{c} z^{2} \end{array}}{\begin{array}{c} {\left(\mathrm{cl}\right)}^{2} \end{array}}=1.$$

**Figure 2:**
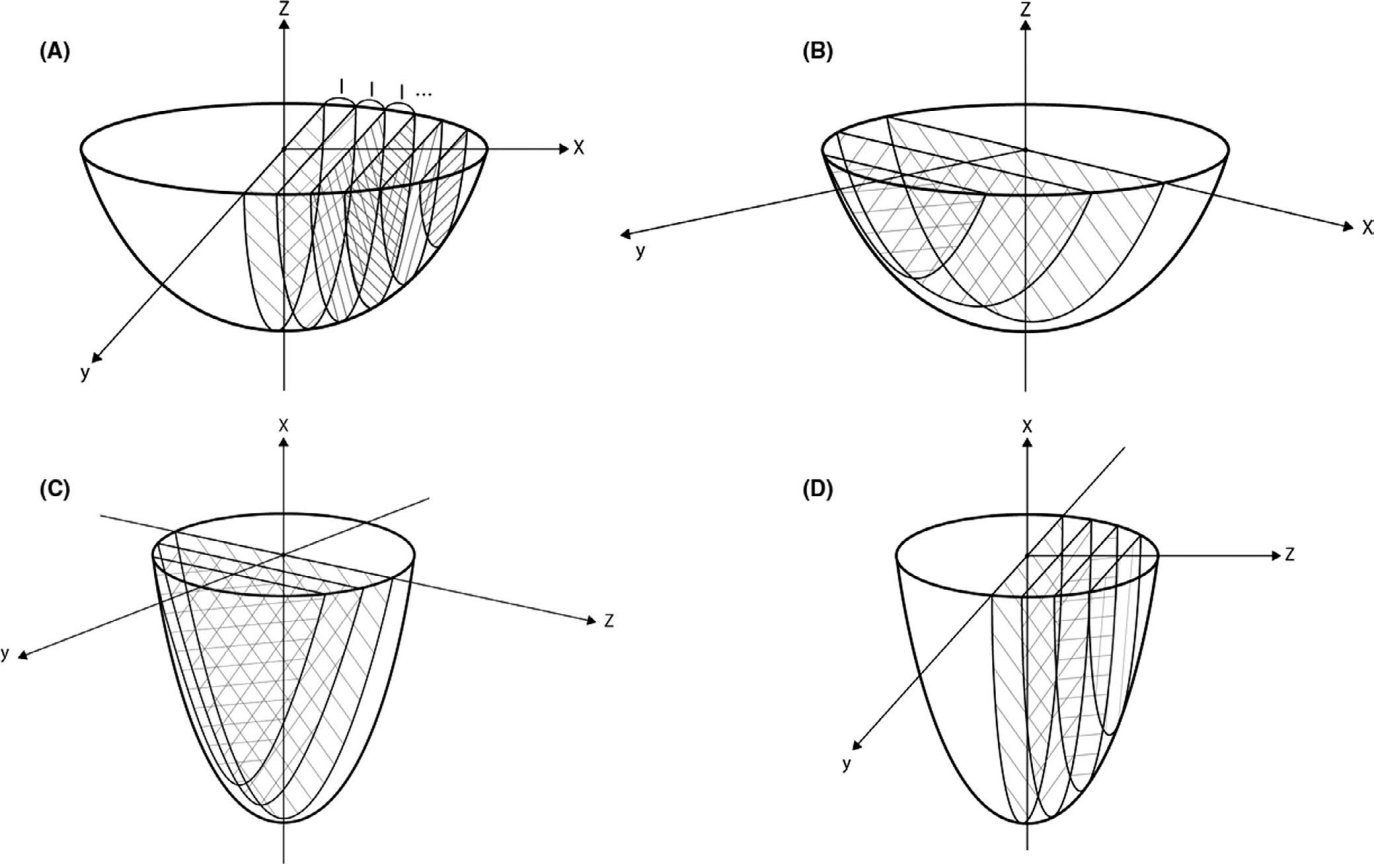
Schematic illustrations of (A) a longitudinal incision on the tunica albuginea and transverse slicing incisions in the parenchyma, (B) a longitudinal incision on the tunica albuginea and sagittal incisions in the parenchyma, (C) a transverse incision on the tunica albuginea and sagittal incisions in the parenchyma, and (D) a transverse incision on the tunica albuginea and coronal incisions in the parenchyma. The width of the sliced piece of testicular parenchyma was assumed to be *l*

Using this testis model, we simulated microTESE using various methods. Regarding the initial incision on the tunica albuginea, two types of incision are possible: longitudinal or transverse. The initial incision on the tunica albuginea would produce a bivalved testis, and the tunica albuginea could be pushed upward turning the testicular parenchyma inside out. Then, the parenchyma could be divided into several thin‐sliced pieces. Several directions are possible for the cutting planes through the testicular parenchyma: transverse, sagittal, and coronal planes. We excluded the example of a longitudinal tunical incision with coronal parenchymal incisions as well as a transverse tunical incision with transverse parenchymal incisions, which are clinically impossible because the divided pieces of testicular parenchyma would be detached from the tunica albuginea and fall apart from the testis in these two examples. Thus, we assumed four types of methods: longitudinal incision on the tunica albuginea and transverse incisions in the parenchyma (Figure 2A), longitudinal incision on the tunica albuginea and sagittal incisions in the parenchyma (Figure 2B), transverse incision on the tunica albuginea and sagittal incisions in the parenchyma (Figure 2C), and transverse incision on the tunica albuginea and coronal incisions in the parenchyma (Figure 2D). We calculated the estimated surface areas for each method and compared them to find the best method for maximizing the cut surface.

### 2.2 NOA patient selection

Diagnoses of NOA were confirmed with several semen analyses by centrifugation as well as reduced testicular volume and increased serum follicle‐stimulating hormone level. Patients with any sign of obstructive azoospermia were excluded from this study. Patients with Y chromosome microdeletions of AZFa and AZFb were also excluded. All patients were counselled pre‐operatively, and all gave written informed consent to perform the surgery.

### 2.3 Application to new NOA patients

We performed microTESE under local anesthesia. The larger of two testes was delivered first through an incision in the median raphe. Under the operating microscope, the tunica albuginea was incised, taking care to avoid equatorial testicular vessels. The testicular parenchyma was examined at a magnification of 10×–20× under the operating microscope. Each sample was taken from the dilated, white tubules and examined by one experienced embryologist in the operating room under a phase‐contrast microscope at 200× magnification. If there were no spermatozoa identified, the contralateral testis was explored. The procedure was terminated when enough spermatozoa were obtained or all regions of both testes had been examined and tubules with the most suitable appearance had been excised. The number of biopsies examined ranged from 1 to 28 per testicle.

From 2011 to 2014, we used a previous method in which the tunica albuginea was incised transversely, and the parenchyma was opened and exposed as a bivalved testis. We searched initially on the cut surface and then in deeper regions of the testicular parenchyma but not systematically.

From 2014 to 2018, we adopted our new method of microTESE, which was developed using the findings obtained from the 3D simulation of microTESE. In this method, the tunica albuginea was incised longitudinally, and Mosquito clamps were placed on each respective side of the tunical incision. The testicle was incised and opened to form a bivalved testis, and the tunica albuginea was pushed upward with gentle pressure to turn the parenchyma of the testicle inside out. Then, the parenchyma was divided transversely into several slices, the width of which was as thin as possible (2‐3 mm).

All procedures were performed by one experienced surgeon (KI), who had performed more than 200 procedures of microTESE prior to 2011. Institutional review board approval (No.201336) was obtained for this study.

### 2.4 Follow‐up after microTESE

All patients were prescribed 10 tablets of loxoprofen (60 mg) and three diclofenac suppositories after the operation, and were informed that additional pain killer was available if necessary. Post‐operative follow‐up visits were scheduled at 1 week, 3, 6, 9, and 12 months after the operation. Post‐operative complications, including wound infection, hematoma formation, and physical or mental symptoms related to androgen deficiency, were checked at every follow‐up visit.

### 2.5 Data analysis

We retrospectively collected data from the medical charts of patients who underwent the previous method between 2011 and 2014, and from 2014, we prospectively followed patients who would undergo the new method. Pre‐operative basic characteristics of patients were compared between both groups, including serum luteinizing hormone, follicle‐stimulating hormone, and testosterone. Sperm retrieval rates, occurrence rates of post‐operative complications, and additional use of pain killer were also compared. Inter‐group differences of categorial, continuous variables were analyzed with chi‐square test and Mann‐Whitney U test, respectively. Statistical significance was considered at *P* < .05, and analysis was performed using JMP^®^ 14 (SAS Institute Inc).

## 3 RESULTS

### 3.1 3D simulation of microTESE

1) Example with a longitudinal incision on the tunica albuginea and transverse incisions in the parenchyma (Figure 2A).

Area of cut planes of the bivalved testis is:

$$2abl^{2}\pi .$$

Surface area of transverse sections at X=lx is:

$$S_{x}=\frac{\begin{array}{c} bcl^{2}\left(a^{2}-x^{2}\right) \end{array}}{2a^{2}}\pi .$$

Thus, the sum of the surface area of all transverse sections is:

$$\sum_{x=-\left(a-1\right)}^{a-1}4S_{x}=\frac{2bcl^{2}\left(2a+1\right)\left(2a-1\right)}{3a}\pi .$$

Total searching area in this example is:

$$2abl^{2}\pi +\frac{2bcl^{2}\left(2a+1\right)\left(2a-1\right)}{3a}\pi .$$

2) Example with a longitudinal incision on the tunica albuginea and sagittal incisions in the parenchyma (Figure 2B).

Area of cut planes of the bivalved testis is:

$$2abl^{2}\pi .$$

Surface area of transverse sections at Y=ly is:

$$S_{y}=\frac{\begin{array}{c} cal^{2}\left(b^{2}-y^{2}\right) \end{array}}{2b^{2}}\pi .$$

Thus, the sum of the surface area of all sagittal sections is:

$$\sum_{y=-\left(b-1\right)}^{b-1}4S_{y}=\frac{2cal^{2}\left(2b+1\right)\left(2b-1\right)}{3b}\pi .$$

Total searching area in this example is:

$$2abl^{2}\pi +\frac{2cal^{2}\left(2b+1\right)\left(2b-1\right)}{3b}\pi .$$

3) Example with a transverse incision on the tunica albuginea and sagittal incisions in the parenchyma (Figure 2C).

Area of cut planes of bivalved testis is:

$$2bcl^{2}\pi .$$

Surface area of longitudinal sections at Y=ly is:

$$S_{y}=\frac{\begin{array}{c} cal^{2}\left(b^{2}-y^{2}\right) \end{array}}{2b^{2}}\pi .$$

Thus, the sum of the surface area of all sagittal sections is:

$$\sum_{y=-\left(b-1\right)}^{b-1}4S_{y}=\frac{2cal^{2}\left(2b+1\right)\left(2b-1\right)}{3b}\pi .$$

Total searching area in this example is:

$$2bcl^{2}\pi +\frac{2cal^{2}\left(2b+1\right)\left(2b-1\right)}{3b}\pi .$$

4) Example with a transverse incision on the tunica albuginea and coronal incisions in the parenchyma (Figure 2D).

Area of cut planes of bivalved testis is:

$$2bcl^{2}\pi .$$

Surface area of transverse sections at Z=lz is:

$$S_{z}=\frac{\begin{array}{c} abl^{2}\left(c^{2}-z^{2}\right) \end{array}}{2c^{2}}\pi .$$

Thus, the sum of the surface area of all coronal sections is:

$$\sum_{z=-\left(c-1\right)}^{c-1}4S_{z}=\frac{2abl^{2}\left(2c+1\right)\left(2c-1\right)}{3c}\pi .$$

Total searching area in this example is:

$$2bcl^{2}\pi +\frac{2abl^{2}\left(2c+1\right)\left(2c-1\right)}{3c}\pi .$$

5) Comparisons of the above 4 patterns.

1)−2) =

$$2abl^{2}\pi +\frac{2bcl^{2}\left(2a+1\right)\left(2a-1\right)}{3a}\pi -2abl^{2}\pi -\frac{2cal^{2}\left(2b+1\right)\left(2b-1\right)}{3b}\pi =\frac{2cl^{2}\left(a^{2}-b^{2}\right)}{3ab}\pi >0$$

2)−3) =

$$2abl^{2}\pi +\frac{2cal^{2}\left(2b+1\right)\left(2b-1\right)}{3b}\pi -2bcl^{2}\pi -\frac{2cal^{2}\left(2b+1\right)\left(2b-1\right)}{3b}\pi =2bl^{2}\left(a-c\right)\pi >0$$

3)−4) =

$$2bcl^{2}\pi +\frac{2cal^{2}\left(2b+1\right)\left(2b-1\right)}{3b}\pi -2bcl^{2}\pi -\frac{2abl^{2}\left(2c+1\right)\left(2c-1\right)}{3c}\pi =\frac{2al^{2}\left(b^{2}-c^{2}\right)}{3bc}\pi >0$$

**Figure 3:**
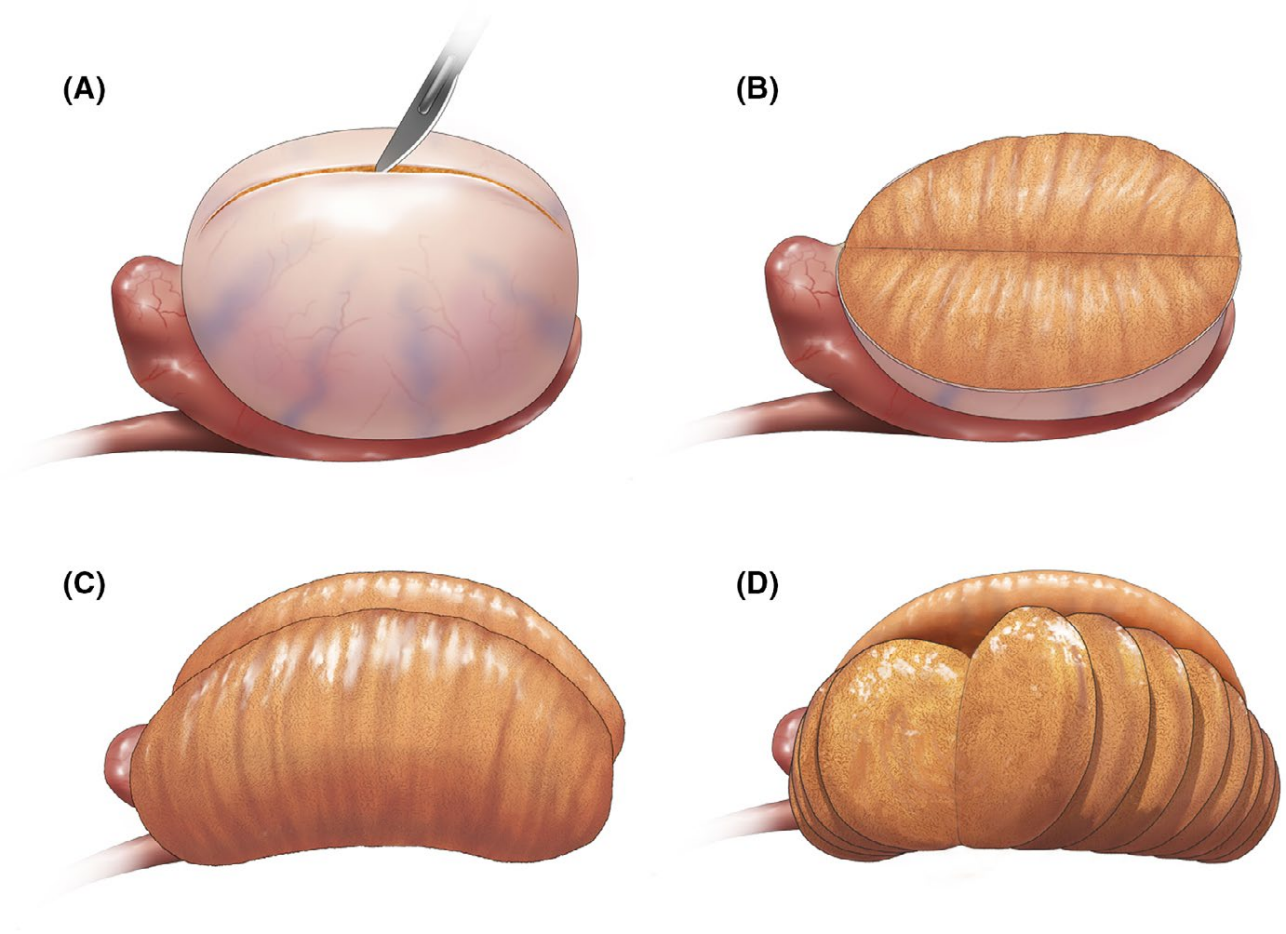
The method to maximize the surface area in microTESE. (A) A long longitudinal incision was made in the tunica albuginea. (B) After the initial incision, the testis was opened by incision to form a bivalved testis. (C) The tunica albuginea was pushed upward turning the parenchyma inside out. (D) Transverse slicing incisions on the parenchyma provided the maximum surface area in microTESE

In conclusion, a longitudinal incision on the tunica albuginea and transverse incisions in the testicular parenchyma (Figure 3A‐D) provided the maximum surface area in the simulation of microTESE.

### 3.2 Application of 3D simulation findings to new NOA patients

Pre‐operative basic characteristics of patients are shown in Table 1. From 2011 to 2014, 56 patients underwent microTESE with the previous method, and 102 patients underwent the new method from 2014 to 2018. The overall SRR was significantly higher using the new method compared with the previous method [46/102 (45%) versus 16/56 (29%), respectively]. Patients were divided into subgroups by etiologies and pathologies of NOA, and higher SRRs were observed in Klinefelter syndrome and uniform maturation arrest subgroups (Table 2). As for post‐operative complications, there were no cases of post‐operative hematoma formation, wound infection, nor androgen deficiency symptoms in either group. One case in the previous method group (1.8%) and two cases in the new method group (2.0%) required additional pain killer after the operation, but there was no significant difference between the two groups (Table 3).

**Table 1:** Pre‐operative basic characteristics of 158 patients stratified with surgical methods

	Previous method	New method	*P*
	(2011‐2014)	(2014‐2018)	
Total number of patients	56	102	
Klinefelter syndrome	8 (14.3%)	11 (10.8%)	NS
AZFc deletion	0	2 (2.0%)	
47XYY	0	2 (2.0%)	
Post–anti‐cancer chemotherapy	4 (7.1%)	3 (2.9%)	
History of cryptorchidism	3 (5.4%)	7 (6.9%)	
Others	41 (73.2%)	77 (75.5%)	
Average age (years, range)	37.0 (27‐51)	37.0 (26‐61)	NS
Average luteinizing hormone (mIU/mL, range)	8.1 (2.3‐28.2)	8.0 (1.5‐29.6)	NS
Average follicle‐stimulating hormone (mIU/mL, range)	20.0 (2.9‐55.9)	21.4 (2.7‐61.9)	NS
Average testosterone (ng/mL)	3.7 (0.4‐12.1)	4.2 (0.7‐9.6)	NS

**Table 2:** Sperm retrieval rates of the overall and the subgroups classified by etiologies and pathologies of NOA

	Previous method	New method	*P*
	(2011‐2014)	(2014‐2018)	
Overall successful sperm retrieval	16/56 (29%)	46/102 (45%)	<.05
Klinefelter syndrome	1/8 (12.5%)	5/11 (45.5%)	NS
AZFc deletion	0/0	1/2 (50%)	‐
47XYY	0/0	2/2 (100%)	‐
Post–anti‐cancer chemotherapy	0/4 (0%)	0/3 (0%)	‐
History of cryptorchidism	3/3 (100%)	6/7 (85.7%)	NS
Others	12/41 (29.3%)	32/77 (41.6%)	NS
Hypospermatogenesis	13/24 (54.1%)	32/44 (72.7%)	NS
Uniform maturation arrest	0/2 (0%)	8/9 (88.9%)	.011
Sertoli cell only	3/30 (10%)	6/49 (12.2%)	NS

**Table 3:** Post‐operative complications of each surgical method

	Previous method	New method	*P*
	(2011‐2014)	(2014‐2018)	
Hematoma formation	0	0	NS
Wound infection	0	0	NS
Symptoms of androgen deficiency	0	0	NS
Additional use of NSAIDs	1/56 (1.8%)	2/102 (2.0%)	NS

## 4 DISCUSSION

MicroTESE and intracytoplasmic sperm injection have become a gold standard treatment option for patients with NOA. The underlying concepts for microTESE are that seminiferous tubules containing many developing germ cells are likely to be larger and more opaque than tubules without sperm production and that the powered field with surgical microscope is beneficial for the search for these tubules. In 1999, the original description of the microTESE technique included the use of a wide transverse opening of the tunica albuginea.^1^ This group also recommended searches deeper than the initially exposed surface of testicular tissue and noted that the insufficient exploration and limited exposure of tissue adversely affected the efficacy of sperm retrieval.^8^ Ramasamy et al reported in their study of 900 cases with microTESE that 65% of patients with successful sperm retrieval had spermatozoa identified around the initial wide equatorial incision, while the remaining 35% of patients required deeper systematic dissection.^9^

The microTESE surgical technique has had few modifications since the original description. Although we believe that a wide opening in the tunica albuginea is essential for a complete search of the testicle, we could not find any report discussing the best way to initially expose the testicular parenchyma. Furthermore, the approach and types of incisions used to perform deeper systematic exploration remain controversial. To clarify these issues, we examined mathematical 3D simulation models of microTESE and calculated the sum of the surface area of different planes cut into the testicular parenchyma. Our rationale was that the possibility of finding suitable tubules during microTESE was dependent on the sum of the cut surface area of the parenchyma and that maximizing the cut surface area would lead to the highest SRR.

Our mathematical 3D simulation models of microTESE revealed that making a longitudinal incision on the tunica albuginea and then transverse slicing incisions on the testicular parenchyma provided the maximal surface area. We applied this updated method to new patients and confirmed that it improved the SRR and demonstrated its safety. We believe that the increased SRR using our new method supported the idea that the possibility of finding suitable tubules in microTESE depends on the sum of the cut surface area of the parenchyma. With our new microTESE method, almost all seminiferous tubules of the whole testicle can be searched systematically. Thus, we could more readily accept the final outcome of microTESE, including cases in which we could not find tubules containing mature spermatozoa.

In the subgroup analysis, high SRRs for the new microTESE method were observed in Klinefelter syndrome and uniform maturation arrest subgroups. In these cases, suitable tubules containing mature spermatozoa were typically found to be very small, limited in area, and hidden behind other tubules. Our results indicate the need to search all areas and tubules of the whole testis for the best SRR in these cases, and patients with severe spermatogenic failure are likely to benefit from this procedure. In contrast, this method appeared to have no benefit for post‐chemotherapy NOA cases (SRR = 0). However, it is likely that no spermatozoa remain in most patients in the post‐chemotherapy NOA subgroup.

There has been controversy about the type of initial incision for microTESE, especially regarding whether longitudinal or transverse incisions would be better for the blood supply to testicular parenchyma. Gümüs et al examined the histological effects of tunical incisions during TESE in an animal model and concluded that there were no differences in histopathology or scoring between longitudinal and transverse incisions.^7^ Past anatomical studies of intratesticular arteries by Jarow demonstrated that arteries most often enter the testis posteriorly underneath the epididymis at the mid pole, and run inferiorly to the lower pole and then equatorially to the superior‐anterior part of the testis.^10^, ^11^ Thus, transverse resections of parenchyma in our method run parallel to the courses of intratesticular arteries and do not interfere with the blood supply.

Post‐operative complications of microTESE have been examined in past papers.^12^, ^13^, ^14^, ^15^ While several comparative studies with conventional TESE and microTESE concluded that microTESE does not worsen the post‐operative decrease in testosterone compared with conventional TESE,^16^, ^17^, ^18^, ^19^, ^20^ a temporal decrease in testosterone after conventional and microTESE was reported.^21^, ^22^ Our new method of microTESE requires multiple cuts in testicular parenchyma, but the small amount of extracted seminiferous tubules was equivalent to that of the previous method of microTESE. As a result, no patients exhibited post‐operative symptoms of androgen deficiency in our study. Although our study showed the short‐term effects and needs a longer follow‐up, we believe there will be limited effects on testosterone production, and testicular damage or loss will not be a significant risk in our new method.

There were no significant wound‐related complications in our patients. To prevent post‐operative complications, it is important to ensure complete hemostasis and meticulous closure of the wound. We performed microTESE at our outpatient‐based clinic, which had no admission facility, and paid considerable attention to hemostasis and to allow sufficient time for surgical wound closure. In this study, we used a 5‐layer wound closure procedure after microTESE. Sutures were run between the tunica albuginea, tunica vaginalis, Scarpa's fascia, Camper's fascia, and epidermal layers. We think that this procedure contributed to the low post‐operative complication rate in our study.

As for post‐operative pain, our new method was comparable with the previous method of microTESE. We completed the new method of microTESE using local anesthesia alone, and all patients could walk out of our clinic right after the surgery. The patients managed post‐operative pain with NSAIDs prescribed after the surgery, and only a few required additional prescriptions.

In conclusion, our mathematical 3D simulation model highlighted a new way to maximize the surface area of testicular parenchyma in microTESE. Our new method achieved a better SRR and had similar post‐operative complications and level of pain management to those of our previous method of microTESE.

## CONFLICT OF INTEREST

None.

## AUTHORS’ CONTRIBUTIONS

All authors qualify for authorship by contributing substantially to this article. KI and OO developed the original concept of this study. Surgeries were performed by KI, YM, NT, HN, and TG Data collection was performed by YM, NT, and HN Statistical analysis was performed by TG All authors have contributed to the discussion, reviewed the final version of this article, and approved it for publication.
